# CT of acute abdomen in the elderly

**DOI:** 10.1186/s13244-025-01955-1

**Published:** 2025-05-07

**Authors:** Juliette Coutureau, Ingrid Millet, Patrice Taourel

**Affiliations:** 1 Department of Medical Imaging, Lapeyronie University Hospital, Montpellier, France; 2 Desbrest Institute of Epidemiology and Public Health (IDESP), Univ Montpellier, INSERM, Montpellier, France

**Keywords:** Acute abdomen, Elderly patients, Intestinal obstruction, Cholecystitis, Mesenteric ischemia

## Abstract

**Abstract:**

Abdominal disorders represent 10 to 15% of all Emergency Department visits in elderly patients. This educational review focuses on acute abdomen pathologies specific to the elderly and on their imaging patterns and proposes a strategy for performing CT scans in this population. Bowel obstruction is the most common cause of emergency surgery in the elderly with a higher proportion of colonic obstructions, in particular obstructive colorectal cancer and sigmoid volvulus. Concerning abdominal inflammatory processes, such as cholecystitis, appendicitis, and diverticulitis, gangrenous cholecystitis and complicated appendicitis are relatively frequently encountered due to delayed diagnoses. Bowel ischemia, which includes acute mesenteric ischemia (AMI) and ischemic colitis (IC), is also much more common after the age of 80. Although ischemic colitis is mainly related to cardiovascular risk factors, it can also result from a persistent distension above a colonic cancer or from fecal impaction. Finally, extra-abdominal pathologies responsible for acute abdominal pain, such as inferior myocardial infarction, should not be overlooked. In clinical practice, when possible thanks to sufficient and appropriate radiological resources, we recommend a scan without injection of contrast and an injection depending on the results of the unenhanced scan, decided by the radiologist present at the CT scan room during the examination.

**Critical relevance statement:**

CT is critical in the diagnosis and management of patients over 75 years old with an acute abdomen, given the difficulty of clinico-biological diagnosis, the frequency of complicated forms, and the morbidity induced by delayed diagnosis.

**Key Points:**

- The most common site and cause of bowel obstruction in the elderly is large bowel obstruction due to colon cancer.
- Discrepancy between a poor clinical examination and complicated forms on imaging, particularly for inflammation and infections, is responsible for late diagnosis and increased morbidity.
- Ischemia, including of the small bowel, colon, and gallbladder are common cause of acute abdomen in elderly.
- In patients with upper quadrant pain, consider extra-abdominal causes such as pneumonia or myocardial infarction.

**Graphical Abstract:**

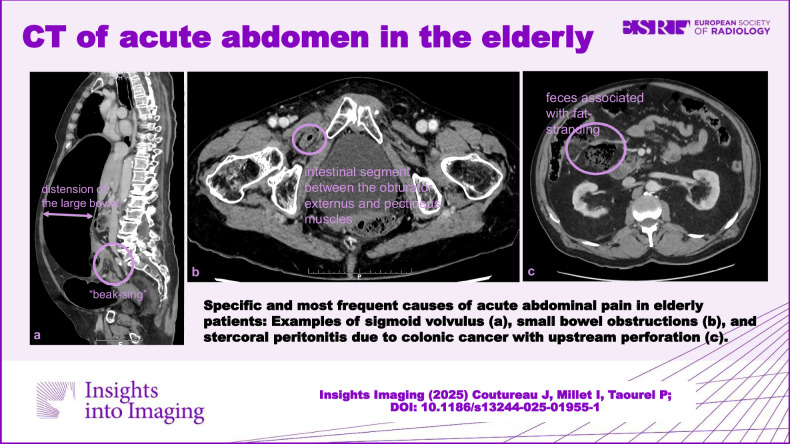

## Introduction

Increasing life expectancy leads to a growing proportion of elderly people in the population. Recent statistical data indicate that 21.3% of the European Union population [1] and 16.5% of the United States population are aged 65 years or older [2]. This demographic shift is expected to result in a corresponding increase in the number of elderly patients with abdominal pain. In the general population, abdominal pain is one of the most common reasons for an emergency department (ED) visit, accounting for approximately 5–10% of all ED visits [3]. This trend is even more pronounced in geriatric patients, in whom a broader category of abdominal disorders constitutes 10–15% of all ED visits, according to audits which included patients aged over 65 [4] or 80 [5]. Although the word “elderly” has conventionally been defined by reference to a chronological age of 65 years or older, epidemiological studies based on physical activity, functional independence, or arteriosclerotic index of the cerebral arteries, have led to a redefinition of “the elderly” as individuals over the age of 75 [6]. These older patients raise special demographic and prognostic issues. In a Finnish audit survey, although patients ≥ 80 years represented only 1.5% of the local population, they accounted for 15% of all ED visits [5]. Similarly, a Japanese administrative database focused on acute abdomen reported that patients aged ≥ 80 years experienced both longer hospital stays and higher in-hospital mortality rates (*p* < 0.001) [7].

It is now well established that CT improves the diagnosis accuracy and the level of diagnostic confidence for disorders responsible for acute abdomen [8] with delay in obtaining results associated with adverse outcomes in older patients [9]. This educational review aims to understand the special expectations and the specific characteristics of CT scanning in a population over 75 years of age for the management of acute abdomen.

## The drawbacks of anamnesis, clinical examination and biological tests

The clinical identification of the cause of acute abdominal pain is particularly challenging in the elderly [10]. The findings of a survey conducted among practicing ED physicians revealed that 78% of the respondents found managing abdominal pain in the elderly more difficult than in younger patients [11]. The preliminary diagnosis at the emergency department was less reliable and hospital mortality was higher in the elderly than in younger patients [12]. Several reasons account for these difficulties including the limited predictive values of simple biological tests in the elderly and clinical pitfalls [13] related to patient’s history and clinical examination.

Factors linked to patient’s history include altered mentation due to fever or electrolyte abnormalities, cognitive impairment, decreased mentation from drugs (e.g., opiates, benzodiazepines), dementia, hearing difficulties, intoxication, language barriers, and psychiatric disorders [13]. Clinical assessment may be impaired [14] by such factors as the absence of fever despite a serious bacterial infection or surgical condition, altered pain perception due to chronic pain medications, coexistent chronic disease(s), lack of hyperthermic response despite a significant intra-abdominal process [15], a lower likelihood of localized tenderness despite a focal surgical condition, reduced rebound and guarding from decreased abdominal wall musculature, suppressed tachycardia caused by medications or intrinsic cardiac disease. In the end, the physical examination cannot reliably predict or exclude significant disease [16].

Concerning biological exams, white blood cells may not be elevated in sepsis or in severe surgical conditions [17]. Liver function tests may be misleading. They are often normal in older adults with cholecystitis, and their elevation does not necessarily indicate acute hepatobiliary disease [18]. Asymptomatic bacteriuria is very common in the elderly and affects women more than men; the incidence is nearly 100% in older institutionalized adults with chronic indwelling Foley catheters. Therefore, although it constitutes the most common cause of abdominal infection in the elderly, acute abdominal pain associated with bacteriuria should not be systematically attributed to urinary tract infection [19].

These limitations explain why the knowledge of the clinico-biological context does not necessarily improve the radiologist’s performance in interpreting a CT scan for acute abdomen in elderly patients [20]. In addition, cognitive impairment, blunted inflammatory response, transportation or financial issues, anxiety, depression, or the fear of losing independence account for the important delays in seeking care among elderly people presenting to the ED [21–23].

## Distribution of causes of acute abdomen and urgent abdominal surgery in elderly patients

Japanese studies have shown the distribution of the causes of acute abdomen in large cohorts [7, 24]. Extensive data were obtained from a national administrative database, developed in a case-mix system project involving 931 participation hospitals (83 academic and 848 community hospitals), which recorded the causes of acute abdominal pain using the International Classification of Diseases and Injuries, 10th Revision [7]. This database included 11 103 patients older than 20 years of age (42.2% men, 57.8% women), with 1681 of them aged 80 or over. The etiology of acute abdominal pain according to the age of the patient is shown in Table 1. Bowel obstruction including ileus and mechanical obstruction, cholelithiasis and cholecystitis, vascular disorder of intestine and fecal impaction were the more common causes in the oldest patients. By contrast, intestinal infection, acute appendicitis, gynecological disease, or colic pain were more frequent in younger patients [7]. The analysis of the causes of emergency surgery for acute abdominal pain according to age was conducted in another Japanese audit [24] including 1310 adult patients of which 682 were 65 years or older. It showed (Table 2) that bowel obstruction and biliary disease accounted for almost two-thirds of emergent surgeries for acute abdominal pain in patients aged 65 or over, whereas appendicitis constituted almost half of the surgeries performed in younger patients. A 7-year experience of a single European center revealed that among patients over 65 across different age groups, complications related to colorectal cancer, diverticular disease and peptic ulcer disease were significantly more common in inpatients ≥ 85 years compared to the age groups of 65–70 and 71–84 years, respectively (Table 3) [25].

**Table 1:** The etiology of acute abdominal pain according to the age of the patient (adapted from [7])

Etiology	80 years or more	60–79 years	40–59 years	20–39 years
	(*n* = 1681)	(*n* = 3144)	(*n* = 2925)	(*n* = 3353)
Ileus	13.0	11.5	7.7	5.0
Cholelithiasis	7.5	8.2	5.5	2.1
Intestinal infection	6.7	6.2	10.5	15.0
Peritonitis	6.0	5.9	6.2	5.8
Constipation	5.0	2.4	1.4	1.5
Vascular disorders of intestine	4.3	3.3	1.6	0.5
Postprocedural disorders of digestive system	3.2	3.4	1.4	0.7
Gastric ulcer	3.0	3.0	4.9	3.0
Acute cholecystitis	2.7	3.0	1.7	0.7
Diverticular disease of intestine	2.6	3.3	5.5	3.6
Colorectal cancer	2.1	2.4	1.2	0.2
Gastritis and duodenitis	2.0	1.9	4.0	3.3
Acute appendicitis	1.7	3.7	6.6	12.6
Acute pancreatitis	1.4	2.6	3.0	1.5
Perforation of intestine	0.7	1.0	0.4	0.0
Neoplasm of uterus or ovary	0.7	0.7	3.1	5.5
Acute cholangitis	0.6	0.9	0.5	0.1
Calculus of urinary tract	0.5	2.3	3.8	3.2
Hernia	0.3	0.5	0.5	0.2
Duodenal ulcer	0.2	0.8	1.6	1.0
Dyspepsia	0.2	0.3	0.8	0.7
Inflammatory disease of uterus or ovary	0.1	0.1	2.3	5.9
Non-inflammatory disease of uterus or ovary	0.1	0.0	0.7	4.2
Diseases associated with pregnancy	0.0	0.0	0.1	4.4
Endometriosis	0.0	0.0	0.5	1.2

The numbers are percentages

**Table 2:** Etiology of emergency surgery for acute abdominal pain according to age (adapted from [24])

Etiology	65 years or older	18–64 years
	(*n* = 682)	(*n* = 628)
Bowel obstruction	305 (45%)	107 (17%)
Biliary disease	135 (20%)	81 (13%)
Acute appendicitis	95 (14%)	296 (47%)
Perforation of intestine	60 (8.9%)	33 (5.3%)
Urinary disease	23 (3.4%)	7 (1.1%)
Bleeding of intestine	10 (1.5%)	2 (0.3%)
Injury	9 (1.3%)	13 (2.1%)
Enteritis	9 (1.3%)	5 (0.8%)
Abdominal bleeding	7 (1.0%)	6 (1.0%)
Wound deficient	7 (1.0%)	2 (0.3%)
Mesenteric vascular disease	7 (1.0%)	2 (0.3%)
Leakage	6 (0.9%)	9 (1.4%)
Aortic disease	4 (0.6%)	1 (0.2%)
Foreign substance	3 (0.4%)	3 (0.5%)
Perforation of uterus	1 (0.1%)	6 (1.0%)
Anal disease	1 (0.1%)	4 (0.6%)
Ectopic pregnancy	0	37 (5.9%)
Testicular disease	0	6 (1.0%)
Ovarian disease	0	4 (0.6%)
Vagina	0	3 (0.5%)
Abdominal tumor	0	1 (0.2%)

**Table 3:** Indication for emergency surgery in the elderly patients (adapted from [25])

Etiology	85 years or older	71–84 years	65–70 years
	(*n* = 169)	(*n* = 562)	(*n* = 255)
Colorectal cancer complications	41 (24.3%)†	97 (17.3%)†	38 (14.9%)†
Acute cholecystitis	31 (18.3%)	145 (25.8%)	64 (25.1%)
Non-malignant bowel obstruction	31 (18.3%)	97 (17.3%)	43 (16.9%)
Complicated diverticulitis	16 (9.5%)†	36 (6.4%)†	8 (3.1%)†
Peptic ulcer disease complications	15 (8.9%)†	36 (6.4%)†	7 (2.7%)†
Acute appendicitis	10 (5.9%)†	41 (7.3%)†	38 (14.9%)†
Acute intestinal ischemia	7 (4.1%)	22 (3.9%)	6 (2.4%)
Other non-malignant*	6 (3.6%)	39 (6.9%)	12 (4.7%)
Peritoneal carcinomatosis	4 (2.4%)	10 (1.8%)	5 (2.0%)
Pancreatic cancer complications	3 (1.8%)	14 (2.5%)	10 (3.9%)
Gastric cancer complications	3 (1.8%)	10 (1.8%)	8 (3.1%)
Other malignant#	2 (1.2%)†	15 (2.7%)†	16 (6.3%)†

* Other non-malignant = trauma/intra-abdominal abscesses requiring surgical intervention

# Other malignant = retroperitoneal sarcoma, ovarian or bladder cancer causing ileus

† *p* < 0.05

## CT patterns of acute abdominal pathologies highly prevalent in the elderly

The pathologies encountered most predominantly in the elderly compared to younger patients include bowel ischemia, sigmoid volvulus, fecal impaction, foreign body perforation, and urinary retention.

## Bowel ischemia

Bowel ischemia, which includes acute mesenteric ischemia (AMI) and ischemic colitis (IC), is ten times more common after the age of 80 than before the age of 60 [26] (Table 1), with a mean age of 80 for acute mesenteric ischemia and 70 for ischemic colitis.

### Acute mesenteric ischemia (AMI)

The incidence of AMI increases exponentially with age [27]. In patients aged 75 years or older, AMI is a more prevalent cause of acute abdomen than appendicitis (Table 2). The typical finding of AMI, characterized by severe abdominal pain disproportionate to physical examination findings, is uncommon in the oldest patients who present with vague abdominal pain, vomiting, and diarrhea [28]. With this symptomatology hardly suggestive of a surgical emergency, the diagnosis often leans towards enteric infection, especially since older patients with infectious enteritis rarely report typical symptoms such as fever or bloody diarrhea [29]. The misdiagnosis of AMI as gastroenteritis is rightly considered a potential pitfall in the elderly [30]. Furthermore, the distribution of the causes of AMI has changed, and in oldest patients, the most common causes are mesenteric arterial thrombosis and non-occlusive mesenteric ischemia rather than embolism [31]. The first challenge in diagnosing AMI is to consider it as a possibility, even in the absence of the typical clinical signs, and to promptly perform a CT scan. Diagnostic delay is known as the primary factor accounting for high mortality rates ranging from 30 to 70% in AMI [32]. CT findings in AMI are summarized in Table 4 [27].

**Table 4:** Computed tomography findings in acute mesenteric ischemia [27]

Vascular findings
Arterial embolus (oval-shaped clot in a previously unaffected artery)
Arterial thrombus (clot with superimposed calcified lesion)
Mesenteric atherosclerosis
Mesenteric venous thrombosis
Portomesenteric venous gas
**Intestinal findings**
Abnormal bowel wall enhancement (decreased, increased)
Bowel wall thickening (edema, hyperdense hemorrhage)
Luminal dilatation (paralysis)
Pneumatosis intestinalis
**Other intra-abdominal findings**
Mesenteric fat stranding (edema)
Ascites
Pneumoperitoneum
Solid organ infarct

**Fig. 1:**
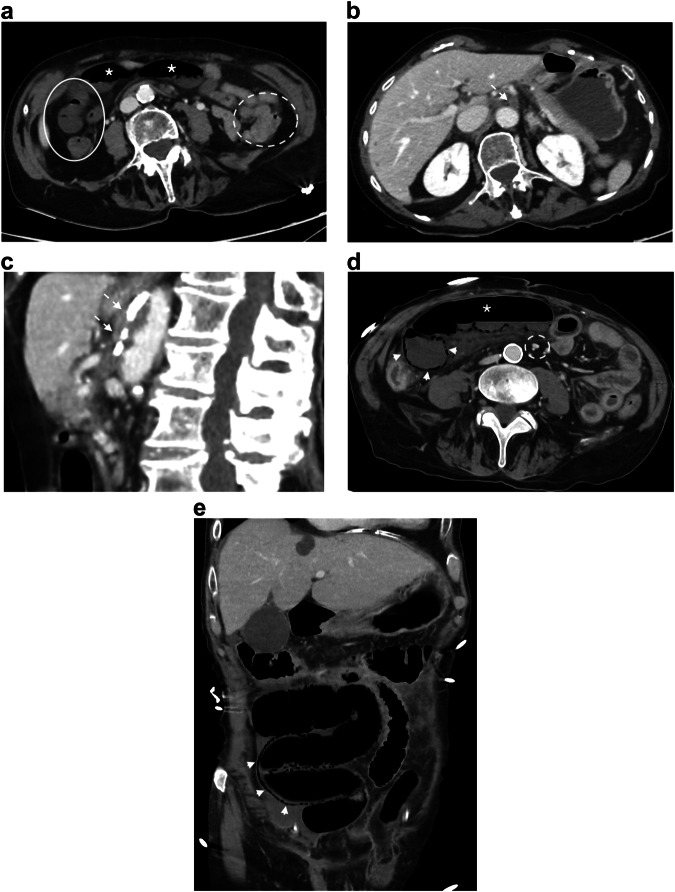
Acute mesenteric ischemia (AMI) due to mesenteric thrombosis and non-occlusive mesenteric ischemia in two different patients. AMI due to thrombosis: axial portal phase CT slice (**a**) in an 88-year-old woman shows decreased enhancement of the small bowel wall (plain circle) compared to another segment (dotted circle). Some of the concerned segments show a thin, “virtual” wall (asterisk). Axial portal phase CT slice at an upper level (**b**) shows a proximal thrombus in the superior mesenteric artery (arrow). Sagittal arterial phase CT (**c**) shows an occlusion of an extensively calcified mesenteric artery (arrows). AMI due to NOMI: axial and coronal portal phases (**d**, **e**) in a 78-year-old man shows dilatation of the small bowel lumen associated with a decreased enhancement of the small bowel wall and parietal pneumatosis (arrowheads). The mesenteric vessels are permeable (dotted circle)

Triphasic CT is recommended, including pre-contrast scans to detect vascular calcifications, hyper-attenuating intravascular thrombus, and intramural hemorrhage; arterial phase to detect thrombus and embolism in mesenteric artery and vein; and portal phase to assess the enhancement of the bowel wall and to look for infarcts of other organs [33] (Fig. 1). Consequently, the second challenge for the diagnosis is to perform an arterial phase study, which is useful to identify mesenteric arterial abnormalities [34]. The third challenge is to consider non-specific findings as possible signs of early ischemic injury, such as mesenteric fat stranding, bowel lumen dilatation, bowel wall thickening, abnormal increased enhancement of the bowel wall, or extensive arterial calcifications [27, 35] and take into account that CT finding of non-occlusive mesenteric ischemia (NOMI) may overlap with other small bowel disease, such as infectious or inflammatory enteritis [36].

### Ischemic colitis (IC)

**Fig. 2:**
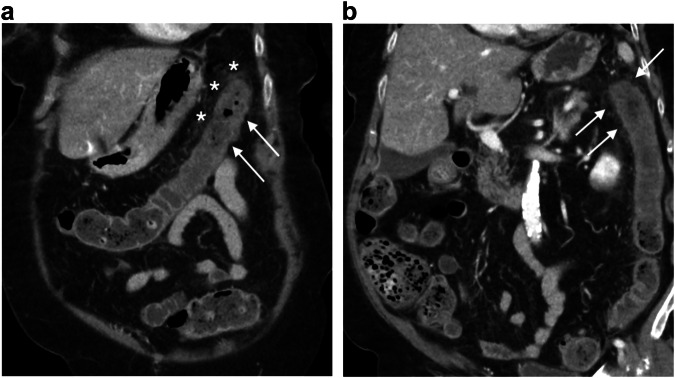
Ischemic colitis. Axial and coronal portal phase CT (**a**, **b**) in a 90-year-old man shows a segmental thickening of the left colonic angle with a stratified enhanced wall (arrows) and peripheric fat stranding (asterisk), suggesting a reperfusion form of ischemic colitis

IC represents the most common cause of intestinal ischemia [37, 38]. Risk factors other than age include hypertension, diabetes, kidney disease, and coronary artery disease [39]. CT is recommended as the imaging modality of choice to be performed within the first few hours of admission [40]. CT features depend on the phase of the IC [41]: in the most common reperfusion form, CT reveals mucosal hyperdensity (the “little rose” sign), a stratified enhanced wall (target or double halo), and pericolic fat stranding. In cases of arterial-ischemic damage, CT shows a thin and unenhanced colic wall, a gas-filled dilated colon, and sometimes colic pneumatosis and pneumoperitoneum. In both forms, the segmental involvement is a diagnostic clue (Fig. 2). There is no specific CT pattern based on the age of the patient [42], even though age is a predictive factor of mortality after colectomy in IC [43].

**Fig. 3:**
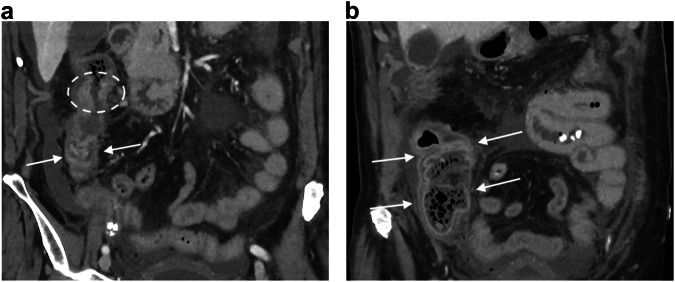
Ischemic colitis complicating a right colonic cancer. Axial and coronal portal phase CT (**a**, **b**) in an 82-year-old woman shows a stratified enhanced thickening (arrows) upstream a short and irregular unstratified thickening (dotted circle) of the right colonic wall

IC can result from colon cancer and appears on CT as smooth, annular wall thickening with a homogeneous or layered enhancement pattern that is frequently contiguous with an irregularly thickened tumorous segment (Fig. 3) [44].

## Sigmoid volvulus

**Fig. 4:**
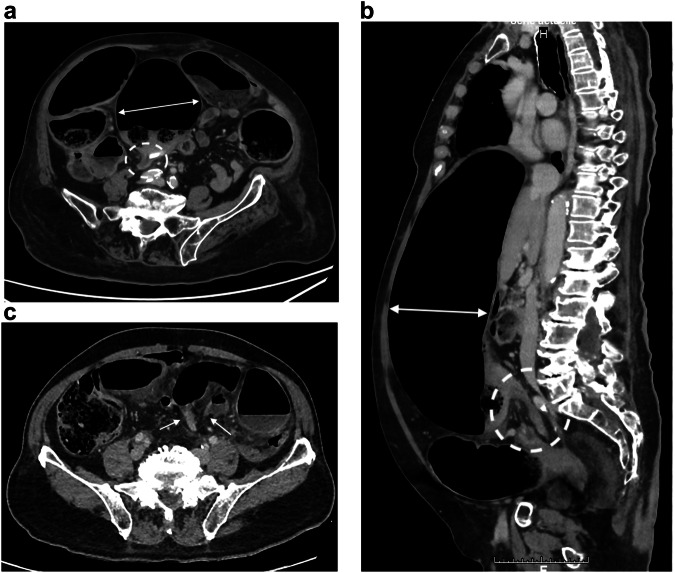
Volvulus of the sigmoid. Axial and sagittal portal phase CT (**a**, **b**) in an 86-year-old woman shows distension of the large bowel (arrows) above a unique “beak-sign” (dotted circle) corresponding to the transition point of an organo-axial sigmoid volvulus. **c** Volvulus of the sigmoid. Axial portal phase CT (**c**) in a 91-year-old man shows a mesenteric-axial sigmoid volvulus as suggested by the presence of two adjacent “beak-signs” (arrows)

Sigmoid volvulus occurs more commonly in the elderly, particularly in individuals with dementia or a psychiatric illness because of a sedentary lifestyle and chronic constipation due to medication, and it is associated with severe comorbidities in one-third of the patients [45, 46]. Classically, diagnosis is initially based on plain abdominal radiographs, which may theoretically be sufficient with CT used in cases of diagnosis uncertainty [47]. However, in clinical practice in patients with suspicion of obstruction, CT is always used since, in addition to providing the anatomic detail of the bowel obstruction site, common findings at CT include identifying the mechanism of the volvulus, i.e., mesenterico-axial pattern versus organo-axial pattern (Fig. 4) and the “whirl” sign of twisted mesenteric vessels [48]. CT may show features of ischemia, predicting surgical over endoscopic intervention and colonic distension greater than 9 cm, which is a risk of recurrent sigmoid volvulus [49].

## Complications of fecal impaction (FI)

FI is defined as a large mass of compacted feces at any intestinal level that cannot be evacuated spontaneously [50]. The disease is highly prevalent in the elderly population. Uncomplicated FI is typically responsible for constipation and rectal discomfort, and the presentation of FI as an acute abdomen is often a result of complications. These complications can be categorized into three main groups based on the damage location: bowel wall, intestinal lumen or adjacent structures [50]:

1. Damages on the intestinal wall are the consequences of the increase of intraluminal pressure over the capillary perfusion, leading to ischemia [50]. They include stercoral colitis, ulcer, and colorectal perforation, with the latter being the most severe complication [50]. CT findings of these ischemic complications include colon wall thickness > 3 mm at stool impaction, spontaneous dense mucosa, mucosal sloughing (dislodged into the lumen), perfusion defect or diffuse lack of enhancement of the colic wall (Fig. 5), pericolic stranding, pericolonic abscess, mottled substances around the damaged colon meaning extraluminal stools, and extraluminal fluid or gas [51]. Dense mucosa, perfusion defects of the colic wall, ascites, or abnormal gas are risk factors for fatal stercoral colitis [51].

**Fig. 5:**
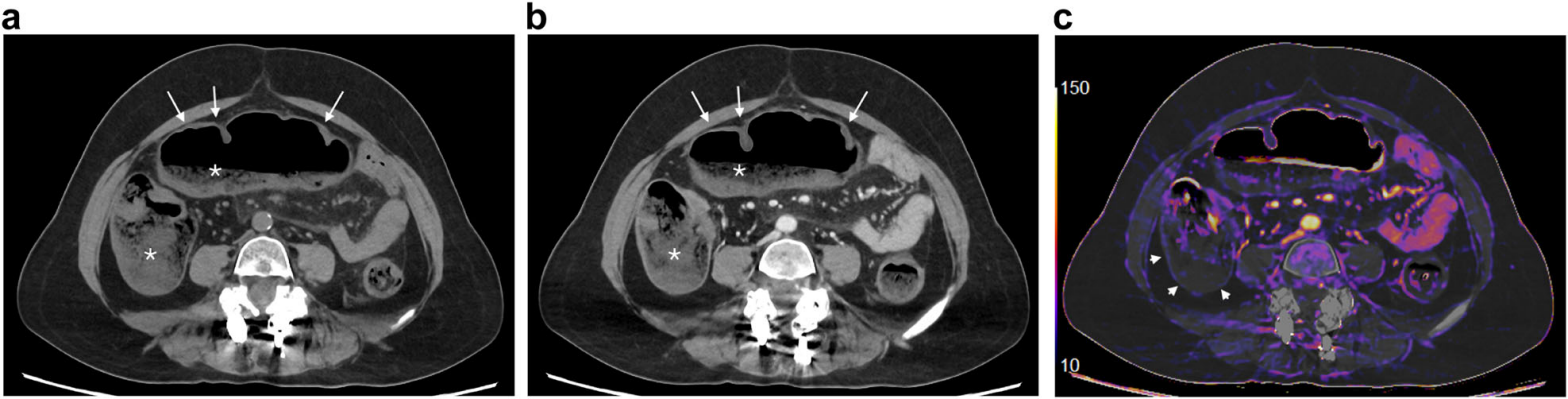
Ischemic colitis complicating fecal impaction. Axial unenhanced and portal phase CT (**a**, **b**) in an 80-year-old woman shows compacted feces in the right and transverse colon (asterisk), associated with a smooth annular wall thickening (arrows), suggesting ischemic colitis due to the distension. Axial subtraction sequence (**c**) confirms a lack of enhancement of the right colonic wall (arrowheads)
2. Damages on the bowel lumen with LBO. CT is useful for identifying the mottled pattern due to feces and for assessing the proximal site of the impaction in order to facilitate management through disimpaction techniques, i.e., digital manipulation, disimpaction under anesthesia, or water-soluble contrast enema for more proximal FI [52]. The continuous contact between feces and wall may cause mucosal irritation, resulting in an increase in mucous secretion and paradoxical diarrhea [50].
3. Damages on the adjacent structures mainly occur on the urinary system with bladder retention and obstructive uropathy [50].

## Perforation due to foreign bodies ingestion

Foreign bodies ingestion is more frequent in the elderly patients because of two predisposing risks: comorbid conditions and the use of dentures, which reduce the sensitivity of the palate [53]. CT will look for a foreign body in the small bowel with localized or free pneumoperitoneum, localized fat stranding, abscess contiguous to the small bowel, or fistula with another segment of the bowel or with the bladder [54].

## Urinary retention

Causes of urinary retention can basically be categorized into obstructive factors (including prostate enlargement), neurologic factors (including medication side effects), and infectious factors [55]. The incidence dramatically increases with age, so that a man in his 80s has a minimum 30% risk of having an episode of acute urinary retention [56]. Although US is both accurate and simple to confirm a bladder retention, this diagnosis is not always considered in elderly patients since they may be unable to provide a clear history. CT, which is often prioritized over US in cases of acute abdomen, will reveal bladder distension and possibly diffuse bladder wall thickening and can identify a cause, which may be an extrinsic compression or a stone within the urethra [57].

## The particularities of the elderly in acute pathologies encountered in the general population

### Bowel obstruction

Bowel obstruction is the most common cause of emergency surgery in the elderly. While Small Bowel Obstructions (SBO) are far more common than Large Bowel Obstructions (LBO) in the general population, older patients proportionally exhibit a significantly higher number of LBOs, particularly obstructive colorectal cancer (Table 3) [25]. Recent studies indicate that the prevalence of emergency surgery for colorectal cancer increases from 11% in patients under 65 to 29% in those over 85 [47, 48, 58].

### LBO due to colon cancer

**Fig. 6:**
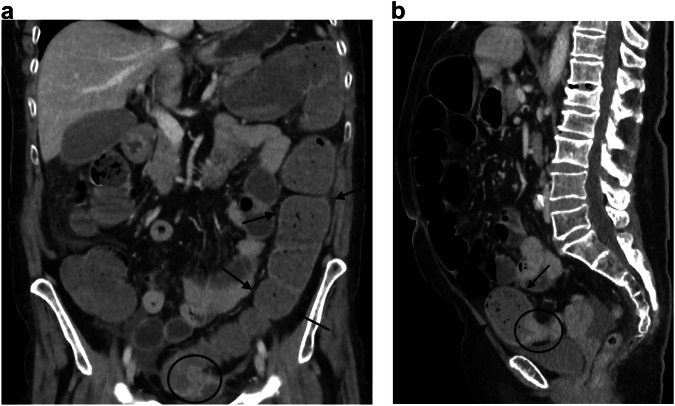
Large bowel obstruction caused by sigmoid cancer. Coronal and sagittal portal phase CT (**a**, **b**) in a 78-year-old woman shows a distension of the large bowel (arrows) above an asymmetric and short-segment thickening of the sigmoid wall (circle) narrowing the colonic lumen

Most acutely obstructive colon cancers occur on the left side, more commonly in the sigmoid colon, because of its narrow luminal diameter [59]. CT findings include asymmetric and short-segment colonic wall thickening or an enhancing soft-tissue mass centered in the colon that narrows the colonic lumen with or without findings of ischemia and perforation (Fig. 6). Obstructing colon cancers often exhibit a shouldering appearance and may be large enough to have central necrosis or, in rare cases, air may be present within the mass, resembling an abscess [60, 61]. Recognition of proximal colonic dilatation aids in identifying the transition point at the tumor site; however, spasm at the splenic flexure in a normal colon can mimic a fixed narrowing [48, 62]. Dilatation of the ascending and transverse colon with distal collapse can be observed in both LBO and chronic colonic pseudo-obstruction [63]. The accurate assessment of obstructive colon cancer is of importance since different options (Hartmann’s procedure, resection with or without primary anastomosis, decompression with proximal colostomy, tube decompression or endoscopic colic stenting) are available [64] for its treatment, depending on the CT results and on the age of the patient. It has been demonstrated in a multicentric national cohort [65], including 3 groups of patients (< 75 years, 75–84 years, and ≥ 85 years) that the oldest patients had higher morbidity with lower survival.

### Small bowel obstruction

**Fig. 7:**
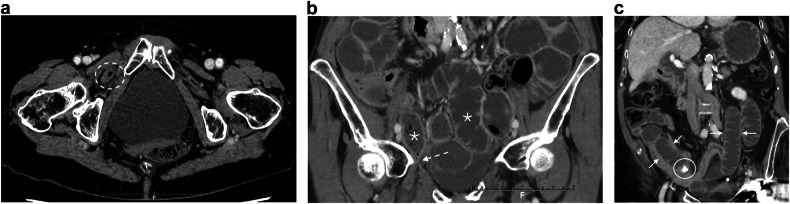
Small bowel obstructions due to causes specifically encountered in the elderly. Obturator hernia in an 85-year-old woman. Axial portal phase CT (**a**) shows an intestinal segment between the obturator externus and pectineus muscles (dotted circle). Coronal portal phase CT (**b**) shows distension of the small bowel (asterisk) up to a transition point corresponding to the collar of the hernia (arrow), suggesting a small bowel strangulation due to an obturator hernia. Biliary ileus in a 90-year-old woman. Coronal portal phase CT (**c**) shows distension of the small bowel (arrows) above a radio-opaque gallstone (circle)

The most common cause of SBO is adhesion in the adult population, whatever the age of the patients [66]. External hernias a relatively common cause of SBO, especially in elderly, due to abdominal wall weakness and conditions which increase intra-abdominal pressure [67]. Among abdominal hernias, obturator hernias, although rare [68], warrant discussion. They affect older women with an average age of 80 [68] and are challenging to diagnose clinically. Although CT is both sensitive and specific for the diagnosis by revealing a mass-like lesion between the obturator externus and pectineus muscles (Fig. 7), some diagnoses are missed in daily practice. Lastly, early surgical intervention is imperative to avoid postoperative morbidity and mortality associated with intestinal strangulation complicating obturator hernia [68].

Among the less common causes of SBO, gallstone impaction primarily affects elderly women, with an average presentation age of 74 years and commonly associated comorbidities [69, 70]. CT easily reveals findings of SBO, air within the gallbladder or the biliary tree, and the presence of an obstructive stone within the bowel lumen (Fig. 7) [71]. By precisely defining the level of the small bowel obstruction, CT can guide enterotomy, which is beneficial for these frail patients [72, 73].

## Inflammatory process

The most common inflammatory processes seen in the elderly are cholecystitis, appendicitis and diverticulitis.

### Cholecystitis and cholangitis

**Fig. 8:**
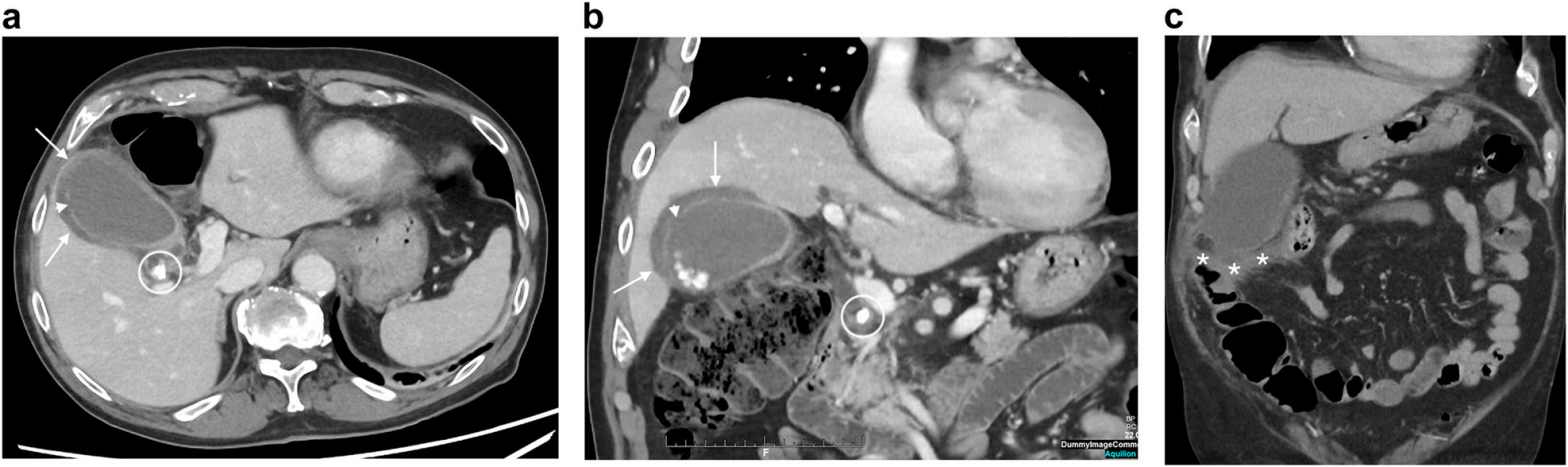
Gangrenous cholecystitis. Axial and coronal portal phase CT (**a**, **b**) in an 80-year-old man shows an irregular thickening of the gallbladder wall (arrows) with interrupted contrast enhancement (arrowhead). The cystic duct is obstructed by a gallstone (circle). Coronal portal phase CT (**c**) in an 81-year-old man shows increased density of fatty tissue around the gallbladder (asterisk) and interruption of the inferior part of the gallbladder wall

**Fig. 9:**
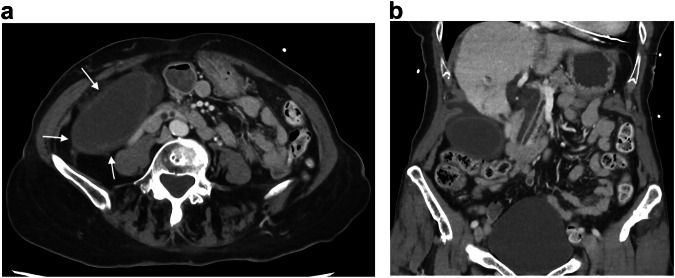
Volvulus of the gallbladder. Axial and coronal portal phase CT (**a**, **b**) in an 84-year-old woman shows dilatation of the gallbladder and horizontal rotation of its axis with poor enhancement of its walls (arrows)

Acute cholecystitis stands as the predominant cause of inflammatory process requiring emergency surgery in the elderly [24, 25] (Tables 2 and 3). In more than 90% of cases, it is due to gallstone, with untreated gallstones generally thought to potentially lead to acute cholecystitis in 10 to 20% of individuals [74]. Age is a strong risk factor of biliary stone and, consequently, of cholecystitis and cholangitis. The epidemiological MICOL study has shown that the prevalence of gallstones in males and females was 15% and 24% at 70 and 24% and 35% at 90, respectively [75]. Ultrasound (US) is recommended as the first-choice imaging method for the morphological diagnosis of acute cholecystitis, because of its non-invasiveness, widespread availability, ease of use, and cost-effectiveness [76] However, there is a growing trend towards the use of CT in cases of suspected cholecystitis [77], particularly in the elderly, where CT scans are performed in half of all cases [78]. There are several reasons for this: CT is widely used in industrialized countries to investigate acute abdominal pain; pre-imaging orientation is more difficult in the elderly; CT permits the diagnosis of an alternative condition or a coincident pathology that would alter therapy; lastly, age is a risk factor for acute gangrenous cholecystitis [79, 80], for which contrast-enhanced CT is recommended [76]. CT findings of gangrenous cholecystitis (Fig. 8) include irregular thickening of the gallbladder wall, poor contrast enhancement of the gallbladder wall (interrupted rim sign), membranous structures within the lumen, (intraluminal flap or intraluminal membrane), gas in the gallbladder lumen or wall, increased density of fatty tissue around the gallbladder, and peri-gallbladder abscess [81, 82]. The diagnosis of gangrenous cholecystitis should lead to emergency cholecystectomy [83]. In the same way, gallbladder volvulus, a rare disease resulting from a twist of the gallbladder along its axis, necessitates prompt surgical intervention because of the risk of ischemia, necrosis, and perforation [84]. Gallbladder volvulus has clinical and physical manifestations similar to acute cholecystitis, making it challenging to distinguish before performing a CT scan. CT may reveal (Fig. 9) dilatation of the gallbladder, displacement of the gallbladder outside its anatomical fossa, rotation of the gallbladder axis from vertical to horizontal, twist along the gallbladder’s vascular pedicle with a swirl sign and abrupt tapering of the cystic duct and ischemic signs on the gallbladder wall [84].

Choledocholithiasis associated with cholecystitis is more common in elderly patients and was encountered in 27% of the 411 patients aged over 80 with cholecystitis [80]. The main risk of choledocholithiasis is cholangitis, characterized by systemic inflammation, cholestasis, and biliary dilatation [85]. Interestingly, while the severity of cholangitis can be classified into three grades (mild, moderate, and severe), age ≥ 75 years is sufficient in itself for grading acute cholangitis as at least “moderate” [85]. Because US has insufficient sensitivity in diagnosing cholangitis and its cause, CT is considered useful for diagnosing cholangitis [85]. CT imaging can clearly identify bile duct dilatation and improve diagnosis of the cause of biliary obstruction (Fig. 8b). Furthermore, it can detect transient hyperattenuation differences in the hepatic parenchyma during the early phase of dynamic CT, which is caused by increased arterial blood flow associated with biliary inflammation [86]. This finding has both diagnostic and prognostic significance. The increase in arterial blood supply usually occurs as a compensatory reaction to a decrease in portal flow, often due to localized hepatic venous obstruction, and was found to be a significant predictor of acute suppurative cholangitis [87]. Finally, CT may diagnose complications, such as liver abscess or portal vein thrombosis [85].

#### Appendicitis

**Fig. 10:**
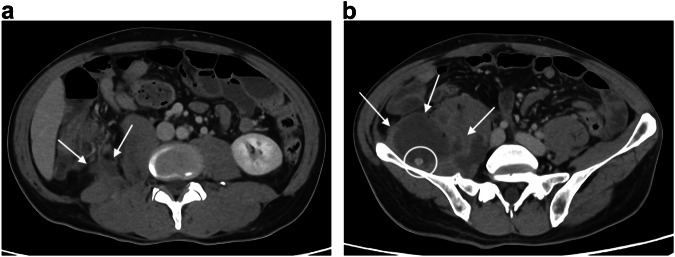
Complicated appendicitis. Axial portal phase CT (**a**, **b**) in a 76-year-old man shows a collection in the right psoas muscle (arrows), in which an appendicolith can be distinguished (circle), suggesting a complicated appendicitis with a psoas abscess

Appendicitis in the elderly is associated with a higher rate of complicated forms, morbidity and mortality, as well as a higher presence of unsuspected appendiceal neoplasms. In a 20-year audit [88], the characteristics of 2060 consecutive patients operated for appendicitis were retrospectively assessed according to age (65 patients ≥ 75 years versus 1995 patients < 75 years). Patients ≥ 75 years had a higher rate of complicated appendicitis (63% versus 13%), higher neoplasm rate on histology (8% versus 1%), higher morbidity (46% versus 8%), greater need for ICU admission after surgery (21.5% versus 0.9%) and higher 30-day mortality (6.2% versus 0.2%). The unreliability of history, physical exam, and laboratory results in the elderly highlights the need for a highly accurate imaging diagnostic test. CT is recommended, due to its superior predictive value in excluding appendicitis and its better reliability in diagnosing complicated appendicitis [89]. The findings to differentiate complicated from uncomplicated appendicitis are well established and include phlegmon, fluid collection, extraluminal appendicolith, periappendiceal air, small bowel dilatation, defect of the appendiceal wall and marked periappendiceal fat stranding [90, 91]. Atypical forms of complicated appendicitis, where the appendiceal structure is not recognized and deep abscesses are present, are more commonly encountered (Fig. 10). In elderly patients, the risk of appendicular tumors must always be kept in mind, when the appendicular diameter is superior to 1 cm with a risk of mucinous neoplasms [28]. In such cases, any medical treatment of suspected appendicitis must be contraindicated, which is why appendicular diameter is a key point in the report [92].

### Diverticulitis

Diverticular disease of the colon, which primarily affects the elderly, occurs in 50–70% of individuals aged 80 years or older [93]. In the remaining population, approximately 5% of all patients with diverticula will develop complications of diverticulitis, such as abscess formation, fistulas, obstruction, or hemorrhage [93]. Age in itself is not associated with complications [93]. By contrast with the general population, right-sided diverticulitis is rare in the elderly: in a study including 223 patients with a right diverticulitis, only 30% of patients (70/223) were over 50 years of age [94]. The clinical presentation of acute diverticulitis is nuanced in the elderly population: only 50% of patients older than 65 years report pain in the lower quadrants of the abdomen, with only 17% exhibiting fever and 43% showing no leukocytosis [95]. In comparison with younger people, a higher proportion of older patients presented with diverticular bleeding [96]. As in the general population, the use of CT scan with IV-contrast is recommended in elderly patients suspected of diverticulitis to confirm the diagnosis and to differentiate between complicated and non-complicated diverticulitis [97]. While ultrasound can be useful in diagnosing diverticulitis, it has limitations, particularly in elderly patients. The accuracy of ultrasound is highly operator-dependent, and it may not be as effective in evaluating the colon compared to CT scans. Additionally, ultrasound has limited ability to assess for complications such as free air or large abscesses, which are crucial in the management of diverticulitis [98]. As clearly pointed out in the American College of Radiology recommendations [99], when suspecting diverticulitis complications, the use of intravenous (IV) contrast material at CT can improve the identification of bowel wall pathology, pericolic abnormalities, vascular pathology, and intra-abdominal fluid collections.

### Bowel perforation

The distribution of the causes of gastrointestinal perforation differs between the general population and elderly patients [53, 100]. Colic perforation due to diverticulitis and cancer constitutes the first two causes of GI perforation in the elderly [53, 100]. Clinical difficulties explain why acute inflammatory, ischemic or tumoral pathologies are more frequently diagnosed at the perforation stage. Colorectal adenocarcinoma constitutes a good illustration of this fact since perforation is more common in older patients. In an epidemiological study on colorectal carcinoma [101], the mean age of the patients who experienced complications was 4 years older than that of those who did not. Conversely, gastric or duodenal perforation occurs in 5 to 10% of elderly patients with ulcer disease with high mortality [102], while the main complication of GI ulcer is bleeding with melena, at least twice as frequent as perforation [102]. On CT images, direct signs of peptic ulcer disease include a focal outpouching and a mucosal enhancement defect, while an actively bleeding ulcer may show intraluminal contrast extravasation [100].

Apart from colon cancer and diverticulitis, the following causes of GI perforations very clearly predominate in the elderly population [53]: perforations due to foreign bodies, bowel ischemia, and stercoral colitis have been discussed above.

**Fig. 11:**
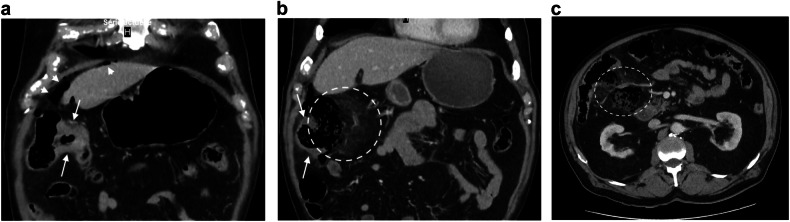
Stercoral peritonitis due to colonic cancer with upstream perforation. Coronal and axial portal phase CT (**a**–**c**) in a 78-year-old man shows free intra-peritoneal gas (arrowheads) and feces associated with fat stranding (dotted circle). The site of the perforation is located above a short-segment thickening of the right colonic wall (arrows), showing a perforation due to distension above a tumoral stenosis

Early detection and treatment of gastrointestinal (GI) tract perforation is crucial for improving patient outcome, particularly in elderly patients due to their frailty [53]. CT is the first-line imaging modality in patients with suspected GI perforations. It plays an important role in assessing the perforation site, in determining the pathology causing the perforation whose prevalence depends on the perforation site, and in anticipating the ensuing complications [53]. Specific CT patterns according to the site and cause of perforation have been extensively described [54, 100]. In inpatients with gastro-duodenal ulcer, CT may show direct signs of peptic ulcer disease, including a focal outpouching and a mucosal enhancement defect, while an actively bleeding ulcer may show intraluminal contrast extravasation [100]. In patients with colorectal cancer perforation, there are two mechanisms underlying perforation [88, 103]: perforation at the cancer site because of tumor necrosis and/or abscess, and diastatic perforation, located proximal to the tumor, most commonly the cecum, induced by the blowing out of the distended proximal colon due to tumor obstruction. In the case of perforation at the cancer site, CT shows irregular colonic wall thickening and infiltrative pericolonic soft tissue with the absence or small amount of free air, whereas diastatic perforation leads to massive pneumoperitoneum [54, 100]. Stercoral peritonitis can complicate both types of peritonitis (Fig. 11).

Otherwise, in elderly patients with advanced obstructive tumors, self-expanding metal stents, which are increasingly used as a bridge to scheduled surgery or as a palliative option, may be the cause of perforation, particularly in patients treated with chemotherapy [104]. Perforation can occur days to months after the stenting of the tumor; CT shows the stent extending through the site of colonic wall disruption.

### Non-abdominal causes of abdominal pain encountered in the elderly population

**Fig. 12:**
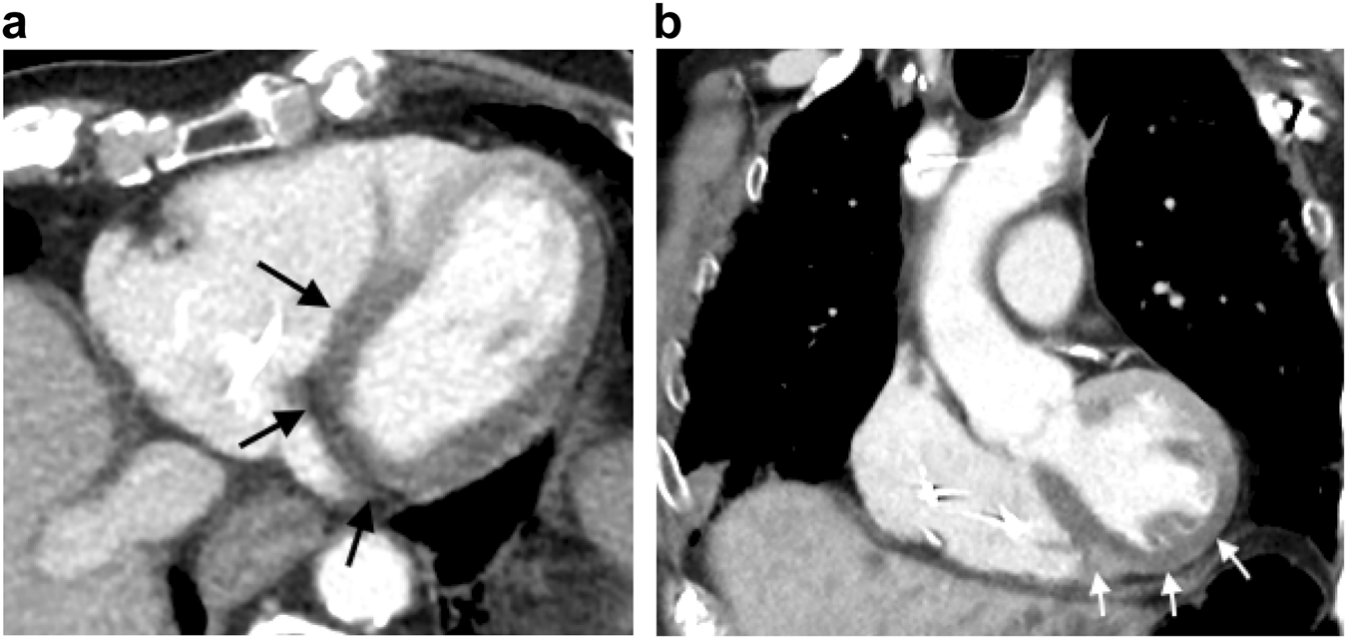
Acute inferior myocardial infarction as a differential diagnosis of acute abdominal pain. Axial and coronal portal phase CT (**a**, **b**) in a 90-year-old man shows a segmental perfusion defect of the inferior myocardia (arrows) corresponding to the territory of the circumflex artery, suggestive of an acute infarction

A common pitfall is overlooking thoracic causes in elderly patients experiencing acute abdominal pain [30]. We will focus more specifically on myocardial infarction for several reasons. It is a common vital emergency and the first cause of mortality in the world; epigastric pain is common in the elderly, particularly among diabetics and patients with inferior infarction [105], with the classic crushing substernal chest pain decreasing in frequency with age. In clinical practice, ECG is inconstantly performed in the emergency room for elderly patients with acute abdominal pain, as shown in a survey-weighted analysis of the National Hospital Ambulatory Medical Care Survey (NHAMCS) [106], where only 39% of older patients with abdominal pain receiving an ECG evaluation. Furthermore, even if performed, both ST-segment elevation may miss and the more common occurrence of left bundle branch block in elderly patients makes ECG interpretation more difficult [107]. The CT pattern of myocardial infarction is characterized by patchy alterations or defects in myocardial perfusion within the affected vascular territory, well seen at the portal phase (Fig. 12) [108]. However, in clinical practice, the diagnosis is often missed. In a recent article involving patients with acute abdomen, who underwent non-gated CT, myocardial infarction was diagnosed in 50% of cases through retrospective reading, compared to only 5% through prospective evaluation [109]. This argues in favor of systematic assessment of myocardial enhancement, even on non-gated acquisition, not properly timed CT-examination to detect non-perfused areas. Obviously, it is unrealistic in these conditions to rely on CT to rule out a myocardial infarction.

### What strategy for the use of CT as triage of elderly patients in the emergency department (ED)?

The strategic application of CT for triaging elderly patients in the ED is a critical consideration. The management of elderly patients presents a significant challenge for emergency physicians, often resulting in extended ED stays. Overnight stays in the ED awaiting ward admission are associated with increased in-hospital mortality and morbidity, particularly among patients with limited autonomy [110]. Given these circumstances, there is a compelling rationale for using CT scans in the clinical triage of elderly patients presenting to the ED with acute abdominal pain.

For optimal imaging of acute abdomen in elderly patients, we use the following CT protocol. The CT scan covers the area from the diaphragmatic domes to the pubic symphysis. A pitch of 0.813 is used to balance image quality and scan speed. The measured slice thickness is fixed at 0.5 mm, with reformatted slices at 3 mm for routine review. The volume of contrast material is adjusted based on the patient’s weight, typically 1 mL/kg (contrast agent used: Iomeron 400). An injection protocol with an initial bolus of 70–100 mL (according to the patient’s weight) of contrast material at a flow rate of 2.5–4 mL/s, followed by a saline flush, is recommended. Scanning is performed during the portal venous phase (80–90 s after injection) and, if necessary, during the arterial phase (30–35 s after injection) to ensure optimal contrast enhancement of the vessel in suspicion of vascular lesion as in acute mesenteric ischemia We use very simple pots processing tools in daily clinical practice: multiplanar reformations in axial, coronal, and sagittal planes are essential for accurate localization and characterization of pathology. The primary window setting used is the soft tissue window, which provides detailed visualization of abdominal organs. Additionally, lung and bone windows can be used to identify pneumoperitoneum and evaluate bony structures, respectively. Techniques such as maximum intensity projection and volume rendering further enhance the assessment of vascular structures and complex anatomical relationships and may be used for communication with the clinical team.

The use of unenhanced CT scans offers two key advantages: it simplifies the triage imaging examination and mitigates the risk of potentially severe iodine-related toxicity in elderly patients. This approach has led to significant improvements in diagnostic accuracy and patient management compared to current practices, as evidenced by two retrospective studies focused on patients over 75 years of age [111, 112]. However, a study by Shaish et al, including a population with an average age of 50, concluded that unenhanced CT was approximately 30% less accurate than contrast-enhanced CT in evaluating acute abdominal pain in the ED [113]. In clinical practice, even when non-contrast scans are extensively used, a contrast-enhanced scan may be necessary if the non-contrast scan fails to yield a positive diagnosis. The primary concern lies in the negative predictive value of a so-called normal non-contrast scan for certain diagnoses, such as bowel or gallbladder ischemia. The strategy of analyzing unenhanced CT before performing a potential injected CT requires evaluating each patient when they are on the table in CT suite, which may not be practical in a busy emergency CT unit. However, this is the prerequisite for personalized medicine, i.e., in this specific case adapted to the patient’s age, the potential adverse effects of iodine injection and the data from the non-injection CT scan. Therefore, if possible, we recommend a non-contrast scan and an injection of contrast media depending on the results of the unenhanced scan, decided by the radiologist present during the examination. Intravenous injection of contrast medium depends on clinical presumption and results of non-injection CT scan. If ischemia of the small intestine, colon, or gallbladder is suspected, the injection is systematic to assess the enhancement of the digestive or vesicular wall and to look for mesenteric vascular obstruction. If a digestive pathology is identified, such as a mechanical obstruction, the injection will enable the search for complications, such as ischemia, and allow a more precise assessment of the cause of the obstruction. On the other hand, in the case of some diseases, such as uncomplicated appendicitis or uncomplicated diverticulitis identified on unenhanced CT, or in the case of a strictly normal CT scan with no clinical suspicion of ischemia, a CT scan without injection may be sufficient. If there is a gap between a very busy scanner and a scarce radiological resource preventing the radiologist from being present in the CT scan room, an injected scan should be performed straight away. There are valid arguments for preferring not to inject iodine in the elderly: the high prevalence of renal failure and the risk of contrast-induced acute kidney injury (CI-AKI). Chronic kidney disease is common in older people, and its prevalence increases in parallel with age exceeding 25% in patients over 70 years old [114] and the risk of CI-AKI is increased, reaching 13.6% in patients over 65, with a 2.55 pooled odds ratio, as calculated in a met analysis recently published [115]. Further studies are therefore necessary before recommending the systematic use of unenhanced CT for triaging elderly patients with suspected acute abdomen. These studies could be structured around a two-stage research strategy: first, the assessment of the negative predictive value of a normal injection-free CT scan, and second, the conducting of a prospective randomized study, to compare the strategy of a systematic injection-free CT upon arrival at the ED with a conventional strategy, which involves on-demand CT scans, usually with contrast injection.

## Abbreviations

AMI: Acute mesenteric ischemia
CI-AKI: Contrast-induced acute kidney injury
ED: Emergency department
FI: Fecal impaction
GI: Gastrointestinal
IC: Ischemic colitis
LBO: Large bowel obstructions
NOMI: Non-occlusive mesenteric ischemia
SBO: Small bowel obstructions
